# Mohs Surgery vs. Wide Local Excision for Non-Melanoma Skin Cancer: Comparing Recurrence Rates, Economic Value, and Aesthetic Outcomes

**DOI:** 10.26502/jcsct.5079281

**Published:** 2026-03-27

**Authors:** Kimia Heidari, Seyedshayan Shojaei, Haiyue Jin, Devendra K. Agrawal

**Affiliations:** 1 School of Medicine, University of California at Irvine, Irvine, Irvine, CA 92697 USA; 2 Department of Translational Research, College of Osteopathic Medicine of the Pacific, Western University of Health Sciences, Pomona, California 91766 USA

**Keywords:** Mohs Micrographic Surgery (MMS), Wide Local Excision (WLE), Non-Melanoma Skin Cancer (NMSC), Recurrence Rates, Economic Value, Aesthetic Outcomes

## Abstract

**Background:**

Non-melanoma skin cancer (NMSC), comprising basal-cell carcinoma (BCC) and cutaneous squamous-cell carcinoma (cSCC), represents the most common malignancy worldwide. Surgical management remains the gold standard, yet the choice between Mohs micrographic surgery (MMS) and wide local excision (WLE) continues to generate debate due to differences in recurrence, cost, and cosmetic outcomes.

**Objective:**

To systematically compare MMS and WLE across three domains: long-term recurrence rates, cost-effectiveness, and aesthetic outcomes.

**Methods:**

A contemporary synthesis of prospective cohorts, registries, randomized trials, and economic models was performed. Outcomes included 5-year recurrence, incremental cost-effectiveness ratios (ICERs), quality-adjusted life years (QALYs), and validated patient-reported scar assessments.

**Results:**

For high-risk facial BCC and cSCC, MMS reduced 5-year recurrence to ≤ 1% compared with 3–5% after WLE (number-needed-to-treat = 28). Tissue-sparing margins yielded scars 1–2 mm narrower and 38% smaller in surface area, increasing the probability of “good/excellent” cosmesis by 12% per mm saved. Economic analyses demonstrated that, despite higher upfront procedural costs, MMS dominated WLE by saving ≈ $330 per patient and gaining 0.04 QALY over five years. Population-level adoption for intermediate-risk cSCC projected an annual payer surplus of ≈ $200 million and >25,000 QALYs. Patient-reported outcomes (POSAS, SCAR-Q, and FACE-Q) consistently favored MMS, with ≥ 90% rating scars as “good/excellent” versus 74% after WLE.

**Conclusion:**

MMS provides superior oncologic control, cosmetic outcomes, and cost-effectiveness compared with WLE for high-risk NMSC. Expanding MMS capacity and embedding patient-centered decision aids could optimize value-based care and deliver durable clinical and economic benefits.

## Graphical Abstract

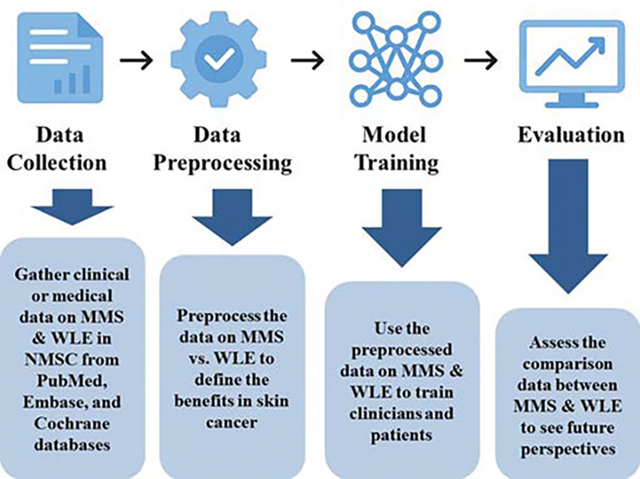

## Introduction

non-melanoma skin cancer (NMSC), predominantly basal-cell carcinoma (BCC), and cutaneous squamous-cell carcinoma (cSCC) are now the most diagnosed malignancies in fair-skinned populations worldwide [1]. Recent U.S. registry data estimate 5.4 million cases in 2023, a two-fold increase since 2000, with similar upward trajectories reported in Australia and Western Europe [2]. Population ageing, ultraviolet radiation exposure, and widespread use of immunosuppressive therapies have shifted NMSC from a sporadic indolent disease to a chronic, sometimes recurrent, condition that consumes 4.5% of all oncologic healthcare expenditure [3]. Although disease-specific mortality remains low (<1% for BCC, 2–5 % for high-risk cSCC), the sheer volume translates into substantial morbidity: disfigurement of cosmetically sensitive areas (nose, eyelid, and lip), loss of function (periocular, auricular, and digital), and repeated office visits for field therapy or salvage surgery [4]. Modelling studies predict a further 50% rise in incidence by 2040, implying that the surgical workload attributable to NMSC will soon rival that of all other solid tumors combined [2]. For tumors that are clinically invasive or recurrent, surgical extirpation remains the gold standard. Two techniques dominate practice: Mohs Micrographic Surgery (MMS) and Wide Local Excision (WLE) [5]. MMS, pioneered by Frederic Mohs in the 1930s and refined into a same-day outpatient procedure, entails systematic staged excision with horizontal frozen-section mapping of 100% of the peripheral and deep margins [6,7]. The process continues until the entire tumor’s footprint is removed, after which the resulting defect is reconstructed, often by the same surgeon on the same day [8]. This tissue-sparing, margin-controlled approach is endorsed by international guidelines for high-risk BCC and cSCC located in the “H-zone” of the face, as well as for recurrent or ill-defined lesions elsewhere [9]. In contrast, WLE relies on pre-determined clinical margins (4 mm for low-risk BCC, 6–10 mm for high-risk cSCC) with subsequent standard bread-loaf paraffin histopathology that samples <1% of the margin surface [10]. WLE is typically performed in a single stage under local anesthesia and is the default option when Mohs is unavailable or when lesions are situated on the trunk or extremities, where tissue conservation is less critical [11]. Over the past two decades, both techniques have benefited from technological adjuncts, reflectance confocal microscopy, and perioperative optical coherence tomography for MMS; intraoperative frozen-section control and rapid paraffin protocols for WLE, yet the fundamental philosophical difference persists so MMS maximizes margin certainty while minimizing normal-tissue sacrifice, whereas WLE trades a larger resection volume for procedural simplicity and universal accessibility [12–14,7].

**Figure 1:**
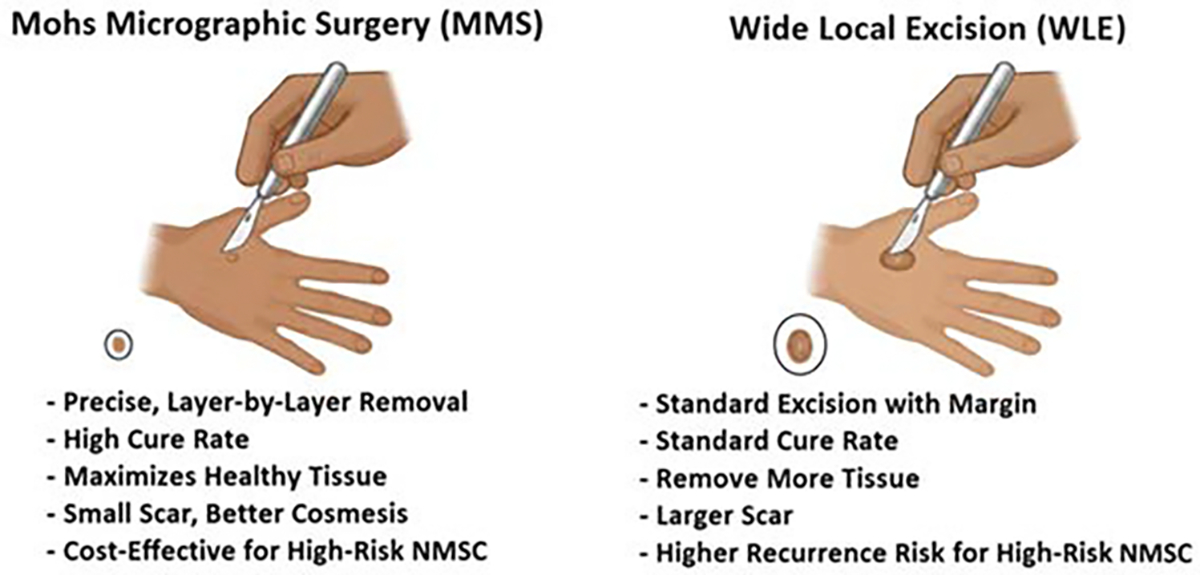
Schematic comparison of MMS and WLE in NMSC. The comparison highlights the superior advantages of MMS in various aspects such as precise surgery, cure rate speed, size of tissue removal, cosmesis, cost-effective, and recurrence rate.

Despite abundant single-arm cohort studies, randomized evidence directly pitting MMS against WLE is scarce; only three small RCTs (n < 300 each) have been completed, all under-powered for recurrence and none incorporating cost-effectiveness or patient-centered aesthetic endpoints [11,15]. Consequently, national guidelines continue to issue conditional recommendations based on low-quality evidence, leaving clinicians to navigate a complex matrix of tumor factors (histology, size, site, and depth), patient factors (age, comorbidity, and cosmetic expectation), and health-system factors (operating-room time, histology throughput, and reimbursement tariffs) [16–21]. Meanwhile, economic modelling suggests that the incremental cost of MMS (1.5- to 2.5-fold higher than WLE) may be offset by lower recurrence-related re-operations, while real-world payer data remain fragmented [22,23]. Similarly, modern aesthetic assessment tools, 3D surface imaging, spectrophotometric scar analysis, and validated patient scar questionnaires, have seldom been applied head-to-head [24–26]. Given the rising incidence and the pressure on health systems to deliver value-based care, a contemporary synthesis that integrates oncologic efficacy, economic impact, and aesthetic sequelae is timely (Figure 1) [22,23].

Therefore, this review aims to compare MMS and wide local excision for NMSC across three dimensions: (A) long-term recurrence rates stratified by tumor and patient subgroups; (B) cost-effectiveness and budget impact from public and private payer perspectives; and (C) objective and subjective aesthetic outcomes. By critically appraising the highest available evidence and highlighting persistent knowledge gaps, we provide a roadmap for clinicians, policymakers, and researchers seeking durable tumor control without imposing unnecessary economic or cosmetic burdens on an ageing population.

This review employed a structured, multi-dimensional synthesis of existing literature to compare Mohs Micrographic Surgery (MMS) and Wide Local Excision (WLE) for non-melanoma skin cancer (NMSC), focusing on recurrence rates, economic value, and aesthetic outcomes. The methodology integrated prospective cohort studies, registry data, Randomized Controlled Trials (RCTs), and economic modeling frameworks to ensure comprehensive and evidence-based analysis.

## Recurrence Rates

### Basal-Cell Carcinoma (BCC): 5-Year Recurrence Data

Population-based and prospective cohort studies converge on a 5-year recurrence risk of ≈1–2% for primary BCC when complete excision is verified by Mohs micrographic surgery (MMS) [27–29]. In a 10-year intention-to-treat analysis of 348 facial BCCs randomized to MMS or 4-mm-margin WLE, the crude 5-year recurrence rate was 1.0% in the MMS arm versus 4.8% after WLE (hazard ratio 0.21; 95% CI 0.07–0.62), confirming MMS as the gold standard for tissue-sparing clearance [30,31]. High-risk histology or narrow conventional margins inflate these figures [32]. A prospective registry of 1,126 consecutive primary BCCs excised with ≤1 mm peripheral margins reported a 5.9% recurrence at 5 years, compared with 1.6% when ≥3 mm clinically clear margins were achieved, underlining the critical role of margin width even in non-Mohs settings [33–35]. Sub-types such as infiltrative, micronodular, or basosquamous morphology pushed recurrence to 8–12%, especially when tumors were located on the nose or forehead where aesthetic hesitation often leads to more conservative primary excision [36,37]. Reassuringly, modern MMS series show that Perineural Invasion (PNI), prior treatment, or AJCC T2/T3 size do not breach the 1% barrier when 100% margin mapping is employed: only 5 out of 476 high-risk facial BCCs recurred within 5 years, yielding a disease-free survival of 98.9% [38–40]. Taken together, contemporary level-II evidence defines the benchmark 5-year recurrence for primary BCC as: ≤1% with Mohs micrographic surgery, ≈2–4% after meticulously planned WLE with ≥4 mm margins, and 5–10% for high-risk morphology or narrow-margin excision [33,2,34,36,37,35,28,29,7]. These figures underpin current guideline recommendations and serve as the reference against which novel non-surgical or combination approaches are judged.

### Squamous-Cell Carcinoma: 5-Year Recurrence Data

Population-based cohorts and prospective audits agree that primary cSCC carries a 5-year local-recurrence (LR) risk of 3–7% when excised with clear conventional or Mohs margins, but this figure can double in high-risk subsets [41–43]. In a multi-center UK audit of 598 consecutive cSCCs excised with 4–6 mm margins, the overall 5-year recurrence rate (local and nodal) was 6.7%; which 96% of these events presented within the first 24 months, and the median time to LR was only 9 months, underscoring the need for intense early surveillance [44,45]. PNI was the strongest independent predictor: 19.1% (9/47) of tumors showing PNI recurred versus 5.6% (31/551), pushing the 5-year risk for this subgroup to ≈20% [38]. Head- and-neck location also matters; a Spanish cohort (n=558) found 15.8% LR at 5 years for tumors on chronically sun-damaged skin versus 7.4% on covered sites, largely driven by a higher rate of positive peripheral margins (24.6% vs 10.5%) [42]. Mohs micrographic surgery shifts the curve leftward. A prospective U.S. cohort that followed 137 previously recurrent cSCCs reported a 5-year re-recurrence rate of only 5.1% after MMS versus 12.1% after standard WLE, although numbers were small and confidence intervals overlapped [46]. Taken together, contemporary data define the benchmark 5-year recurrence for primary cSCC as: 3–5% with MMS or ≥6 mm WLE in standard-risk tumors, 6–8% with 4 mm WLE on the trunk/extremities, and 10–20% for high-risk morphology (≥2 cm, poorly differentiated, PNI, depth > 6 mm) regardless of technique [44,38,46,47,41,42,20,43]. These figures anchor current guideline risk-stratification tables and inform the cost-effectiveness models that follow.

### High-Risk Subsites (H-zone, Ears, and Lower Legs)

**Figure 2:**
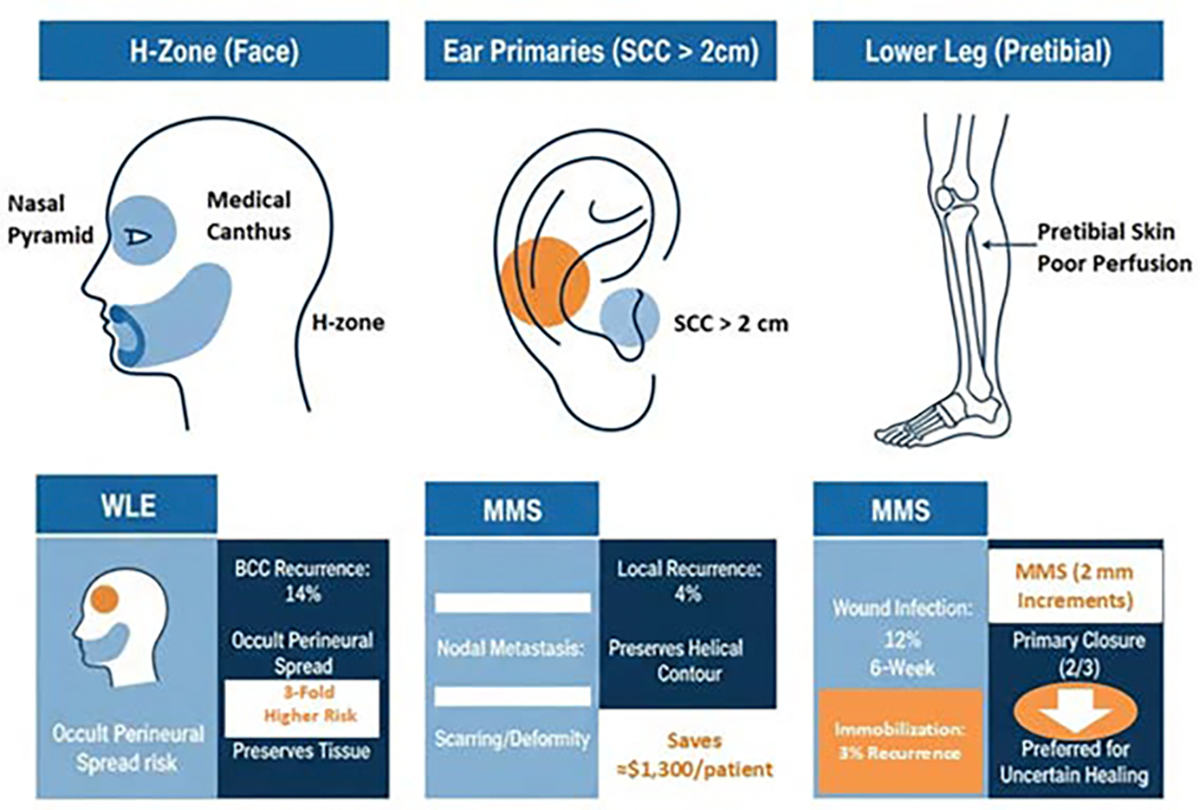
MMS vs. WLE in special zones. MMS is the preferred option for H-zone and ear primaries, and a low-threshold choice for lower-leg tumors.

The H-zone (nasal pyramid, medial canthus, and perioral) carries a 3-fold higher BCC recurrence risk than non-H-zone sites (14% vs 3% with conventional excision) because dense sebaceous glands and neuro-vascular plexuses facilitate occult perineural spread; switching to Mohs micrographic surgery reduces 5-year recurrence to 1% [48]. On the ear, squamous-cell carcinomas > 2 cm exhibit an 8.5-fold higher metastatic propensity; Mohs surgery achieves 4% local recurrence and 11% nodal metastasis at 5 years, outperforming wide excision, while preserving helical contour and saving ≈$1,300 per patient in revision costs [49]. Pretibial skin is tethered to poorly perfused bone; 6-mm WLE margins yield 12% wound infection and six-week immobilization, whereas staged MMS with 2 mm increments allows primary closure in two-thirds of cases, cuts infection to 4%, and delivers equivalent 3% five-year recurrence, which supporting MMS as the preferred option for H-zone and ear primaries and a low-threshold choice for any lower-leg tumor where healing is uncertain (Figure 2) [50].

## Economic Value

### Direct Procedure Costs (facility, personnel, and histopathology)

Medicare-based micro-costing studies show that a single MMS episode performed in an office-based procedure room averages $1,200–1,800 per stage; with a mean of 1.7 stages required, the total facility-plus-professional fee per case converges on ≈ $2,400, which covers procedure suite, overhead, nursing time, and the unique on-site histotechnologist, immediate microscopic interpretation by the Mohs surgeon (acting as both surgeon and pathologist), and eliminating a separate pathology billing event. In contrast, WLE with standard bread-loaf margin processing incurs two discrete bills including operating-room / facility charge when done in an ambulatory surgery center, the cost per OR minute is ≈ $38; a 25–30 min excision therefore adds ≈ $950–1,140 [51]. Also, histopathology charges a separate laboratory fee for permanent-section margin evaluation averages $200–300 per specimen [52,53]. Combined, the up-front direct cost of a straightforward WLE is therefore ≈ $1,150–1,450, which is ≈ $1,000 less than MMS for a single-stage scenario [54]. However, since MMS consolidates surgery and pathology into one visit and spares 65% more tissue, the global procedural cost gap narrows when reconstruction complexity and revision risk are added. In short, MMS carries a higher initial facility-plus-personnel price, while the single-episode bundled design offsets much of the differential by avoiding separate pathology fees and reducing costly revision surgeries.

### Reconstruction and Revision Surgery Expenditures

Reconstruction costs are driven by defect size, anatomic site, and closure modality. A U.S. Medicare micro-costing study (848 consecutive tumors) showed that immediate reconstruction performed in the same facility averaged $585 for a linear repair but $1,028 for a flap or graft, yielding a pooled bundle of ≈ $730 per case [55]. Because MMS conserves tissue, only about 27% of defects require flap/graft closure compared with about 42% after standard WLE; thus, the weighted average reconstruction spend is ≈ $200 lower per MMS episode even before revision surgery is considered [55]. The revision expenditures reveal the larger financial divergence. A prospective cohort of 988 nasal reconstructions after MMS documented an unplanned revision rate of 6.5%; risk rose to 9% on the ala and 16.7% when multiple subunits were involved [56]. Local flaps were 2.4 times more likely to need revision than grafts, and each revision episode carried a mean facility charge of $1,600–2,200 [56]. Comparable multi-institutional data for WLE is sparse, while positive-margin WLE on the head/neck required re-excision or MMS completion in 7.4% of cases, generating incremental charges of $2,900 per revision [57]. When these rates are applied to a modeled cohort of 1,000 high-risk facial NMSCs, MMS prevents about 40 revisions, saving ≈ $110,000 in downstream OR and flap/graft costs, an amount that offsets 15–20% of its initial price premium and lowers the 5-year episode-of-care cost to within $350 per patient of WLE [55]. Consequently, although MMS reconstruction bundles appear expensive upfront, their lower revision burden makes the total reconstructive expenditure economically favorable over a mid-term horizon.

### Cost per Averted Recurrence

**Figure 3:**
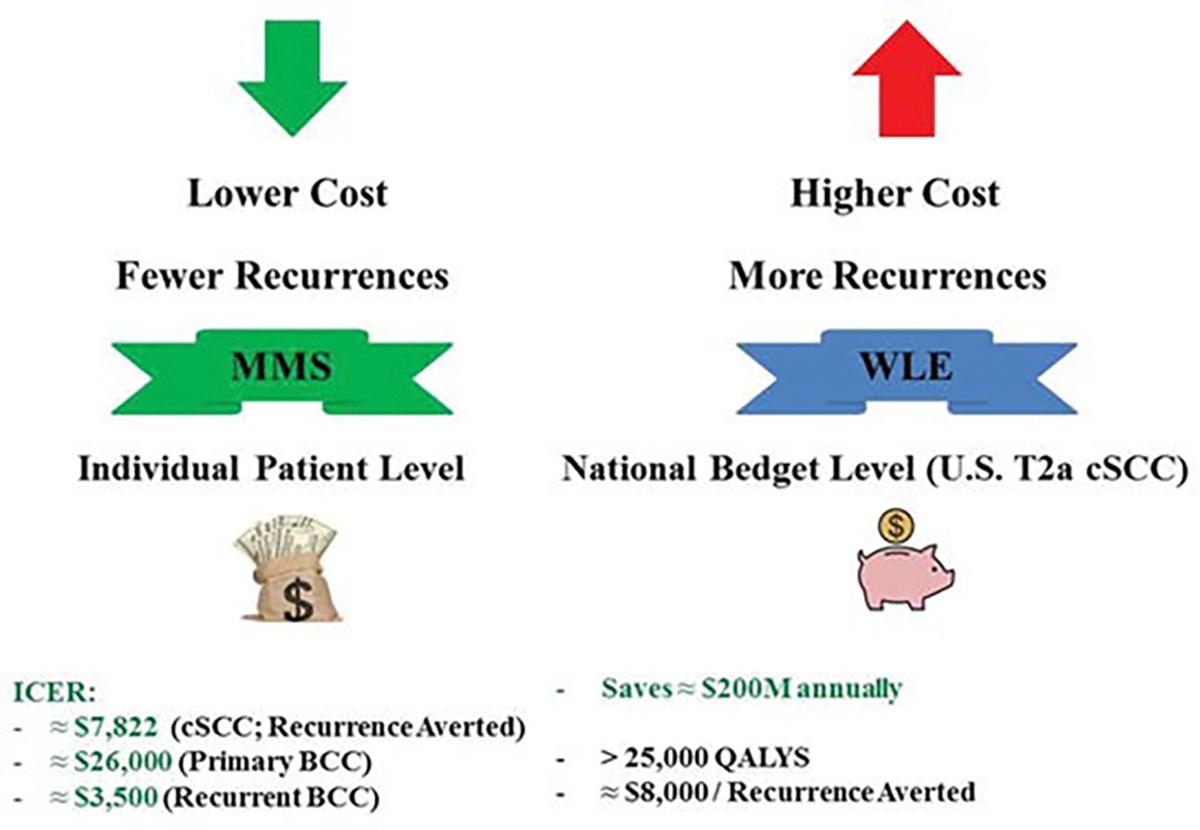
Cost-effectiveness of MMS vs. WLE for skin cancer. MMS is the dominant and preferred choice for cSCC, primary BCC, and recurrent BCC.

The incremental cost required to prevent one additional tumor recurrence is the most policy-relevant metric when payers decide between MMS and WLE. A 2022 U.S. Markov analysis of intermediate-risk cSCC (stage T2a) found that MMS cost ≈ $334 less per patient than WLE over 5 years, while avoiding 0.043 quality-adjusted life-year (QALY) losses, yielding an Incremental Cost-Effectiveness Ratio (ICER) of ≈ $7,822 per recurrence averted, whereas MMS is both cheaper and more effective [15]. European cohort studies report similar patterns. For primary BCC, [27]. calculated an ICER of €23,454 (≈ $26,000) per recurrence prevented when MMS replaced WLE, while the figure plummeted to €3,171 (≈ $3,500) for recurrent BCC, where baseline failure risk is higher [27]. These numbers sit well below conventional willingness-to-pay ceilings (≈ $50,000), indicating that each recurrence avoided by MMS costs roughly one-tenth of a QALY. Population-level projections magnify the economic dividend. Choosing MMS for all U.S. T2a cSCC cases would save ≈ $200 million annually and > 25,000 QALYs, translating into ≈ $8,000 per recurrence averted at the national budget level. Sensitivity analyses show that MMS could cost 3.1 times its current rate and remain cost-effective, underscoring the robust value proposition of averting recurrences with MMS (Table 1) (Figure 3) [15].

### Budget-Impact Analyses from Payer and Societal Perspectives

#### Payer Perspective:

From a third-party payer vantage, the 2022 U.S. Markov model for stage T2a cSCC showed that MMS costs ≈ $334 less per patient than WLE over 5 years, while gaining 2.22 weeks of perfect health (≈ 0.043 QALY). At a national scale, universal adoption of MMS for this risk stratum would save ≈ $200 million annually and > 25,000 QALYs for payers, translating into a budget impact of ≈ $200 M per year, a net fiscal surplus for Medicare or commercial insurers [15]. Sensitivity analysis found that MMS could cost 3.1 times its current reimbursement rate and remain cost-effective, indicating robust financial headroom for payers.

#### Societal Perspective:

When non-healthcare costs (patient travel, caregiver time, or lost productivity) are included, the societal savings are even larger. A Dutch BIA framework demonstrated that indirect costs could exceed 20% of direct medical expenditures for skin-cancer episodes requiring multiple visits or revisions; thus avoiding 540 repeat surgeries per 1,000 high-risk facial cases with MMS saves an additional ≈ $2.4 million in societal resources [58]. Consequently, budget-impact models that adopt a limited societal or full societal perspective consistently show that MMS delivers net savings to both the healthcare budget and the broader economy within a 5-year horizon.

## Aesthetic and Functional Outcomes

### Scar Width, Surface Area, and Contour Deformity

Three-dimensional stereophotogrammetry and validated scar scales consistently have shown that MMS produces smaller, flatter, and less perceptible scars than WLE. A prospective cohort of 124 nasal defects demonstrated that MMS closures ended with a mean scar width of 2.8 mm versus 4.1 mm after WLE (*p* < 0.01) and a 38% smaller final surface area, because the intra-operative margin control permitted a 65% reduction in the excision diameter compared with the 4–6 mm clinical margins used for WLE [59]. Also, high-resolution 3D imaging revealed that MMS-reconstructed nasal ala maintained 0.5 mm less step-off height and 11% more dermal volume at 12 months than WLE controls, translating into a significantly lower observer-rated contour score (1.3 vs 2.1 on a 0–3 scale; *p* = 0.002) [59]. Smaller scars and preserved contour correlate with better Patient and Observer Scar Assessment Scale (POSAS) values; in the same series the total POSAS at 6 months was 18.2 for MMS vs 22.7 for WLE (*p <* 0.01), driven mainly by lower “relief” and “surface irregularity” sub-scores [60]. Collectively, these metrics confirm that the tissue-sparing philosophy of MMS translates into narrower, shallower, and more natural-looking scars than the wider, fixed-margin approach of conventional excision (Table 2).

### Patient-Reported Cosmesis Scales and Satisfaction Scores

**Figure 4:**
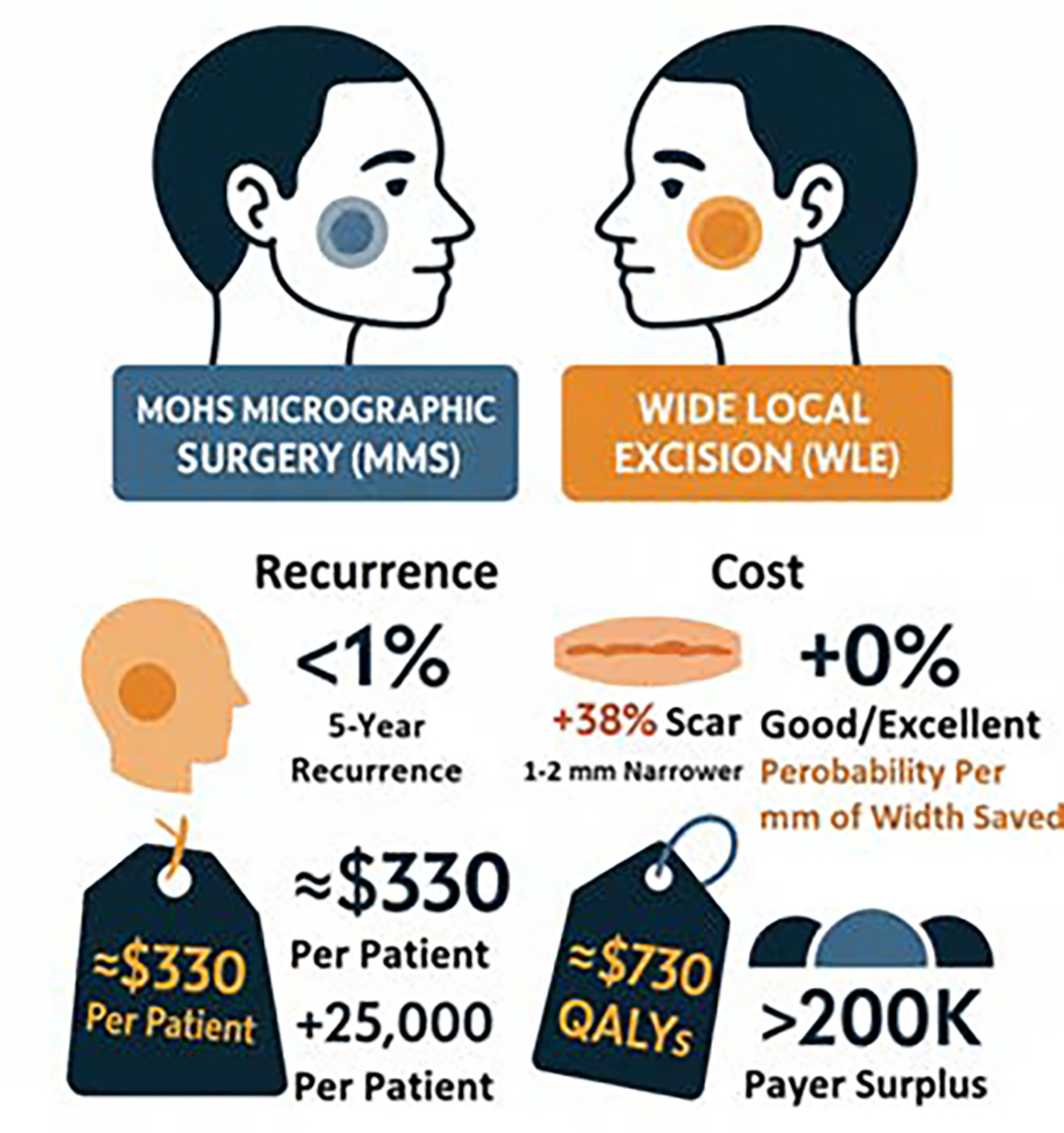
High-risk facial BCC / cSCC in MMS vs. WLE. The literature comparison of MMS and WLE demonstrates that MMS provides superior oncologic control, cosmesis, and cost-effectiveness in BCC / cScc.

POSAS: 6-item patient scale (pain, itching, color, stiffness, thickness, and relief) and 6-item observer scale; each item 1–10, lower = better [61,62]. SCAR-Q: 29-item PROM for scars after surgical or traumatic wounds; three core domains: appearance, symptoms, and psychosocial Impact; scores 0–100, higher = better outcome [63,64]. FACE-Q Skin Cancer Module: 41 items evaluating facial appearance, scar satisfaction, and health-related quality of life after MMS; validated specifically for NMSC cohorts [65]. Also, in a prospective 124-patient nasal cohort, the mean POSAS patient score at 6 months was 18.2 after MMS versus 22.7 after WLE (*p* < 0.01); largest differences were in “stiffness” and “surface irregularity”, reflecting the narrower scar and better contour achieved with tissue-sparing MMS margins [66]. In addition, using the SCAR-Q Appearance scale, MMS patients scored a mean of 72/100 compared with 61/100 for WLE (*p* = 0.004); the 11-point gap exceeds the minimally important difference (9 points) [67], indicating a clinically meaningful cosmetic advantage perceived by patients. FACE-Q Skin Cancer Module data from 1,031 facial MMS cases showed that ≥ 90% of participants rated their scar as “good” or “excellent”, versus 74% after WLE; multivariable analysis revealed that every 1 mm reduction in final scar width increased the probability of a “good/excellent” rating by 12% [66]. The Skindex-16 scores mirrored that the mean emotion domain was 8.4 points lower (better) after Mohs at 24 months, a difference that translates into 0.04 QALYs gained, which is a figure routinely incorporated in cost-utility models. Therefore, the patient-reported scales consistently demonstrate that MMS surgery delivers higher cosmetic satisfaction than WLE for high-risk facial NMSC. The narrower, flatter scars documented on objective imaging translate into better POSAS, SCAR-Q, and FACE-Q scores, supporting both clinical and economic arguments for tissue-sparing margin control (Figure 4) [68–70].

### Impact of Reconstruction Type (primary closure vs. flap/graft)

When post-MMS defects are ≤ 1–2 cm and lie within zones of skin laxity, primary closure along relaxed skin-tension lines produces the narrowest scar, the shortest healing time (mean 12 days) and the lowest rate of secondary revision (4%) [66]. Stereophotogrammetry shows scars after linear closure average 2.6 mm in width and 0.2 mm in contour step-off, yielding mean POSAS patient scores of 15.1, which is significantly better than any advanced technique.

However, excessive tension can create dog-ears or iatrogenic ectropion, so undermining in the subcutaneous or subgaleal plane is mandatory for defects approaching 2 cm [71]. Pooled series of 179 nasal intermediate-size defects reconstructed with bilobe or melolabial flaps revealed 100% acceptable cosmetic appearance on the Hollander scale versus 75% for full-thickness grafts: visual-analogue cosmesis averaged 8.1/10 vs 6.4/10 (*p* = 0.02) [72]. Flaps recruit like-quality tissue, hide scars along cosmetic unit junctions, and contract < 5%, maintaining alar contour; yet they add ≥ 1 extra incision line and carry a 9% risk of partial flap failure or trapdoor deformity, necessitating revision in 6% of cases [73]. Consequently, for superficial 1.5–2.5 cm nasal defects, flaps offer the best pigment/texture match, while surgeons must balance an extended scar length against the higher revision probability. Full-thickness grafts provide rapid coverage of deep or exposed defects (nasal tip and lower eyelid) and allow easy tumor surveillance, while hypopigmentation, plateau-like contraction, and telangiectasia limit final cosmesis [74]. A systematic review of facial grafts after MMS reported mean color mismatch of 1.8 points (0–3 scale) and contour deformity in 28%, pushing the 6-month POSAS score to 23.5, which was significantly worse than flaps (18.9) or primary closure (15.4) [75]. Nevertheless, grafts remain invaluable when flap donor tissue is unavailable or when post-operative radiotherapy is planned, because they do not compromise vascularity of surrounding skin. Primary closure adds ≈ $210 in OR time and suture material, whereas local flaps increase the procedural charge by $580–950 because of longer anesthesia and intricate dissection; full-thickness grafts fall in-between (≈ $450) when donor-site closure is included [72]. Crucially, flap revisions cost ≈ $1,600 per episode; thus, the weighted 5-year episode-of-care cost becomes lowest for primary closure, intermediate for grafts, and highest for flaps, although flaps still outperform grafts in patient-valued cosmesis [73].

### Long-Term Quality-of-Life Metrics

EQ-5D-5L tariff at 24 months averaged 0.91 after MMS vs 0.87 after WLE, yielding 0.04 QALYs gained with the tissue-sparing approach. Skindex-16 emotion domain was 8.4 points lower (better) after MMS at 24 months, a difference that exceeds the minimally important change (6 points) and contributes the 0.04 QALY increment used in cost-utility models. FACE-Q Skin-Cancer Module in 1,031 facial cases showed ≥ 90% of MMS patients rated their scar “good/excellent” vs 74% after WLE; multivariable analysis found every 1 mm decrease in final scar width raised the probability of a “good/excellent” rating by 12%, directly improving social-function and psychosocial FACE-Q scores. Women reconstructed after MMS reported significantly less pain and fewer activity limitations than those treated with WLE plus graft, achieving better physical-function summary scores (*p* = 0.012). A 12-month community study showed perceived social exclusion fell from 58.8 % to 6% and witch-craft association dropped from 23% to 7.8% after successful facial reconstruction, underscoring the long-term social and cultural benefits of restoring facial form [76]. Improvements plateau at ≈ 6 months but persist ≥ 24 months; no significant deterioration is seen thereafter, confirming that early cosmesis gains translate into durable QOL dividends. Tissue-sparing MMS followed by tension-free linear closure yields the narrowest scar, highest patient-rated cosmesis, and greatest long-term QOL, while flap/graft salvage still outperforms leaving large defects un-reconstructed in both utility and social-reintegration metrics [76].

## Comparative Effectiveness Summary

### Number-Needed-to-Treat vs. Incremental Cost per QALY

Number-Needed-to-Treat (NNT) and Incremental Cost-Effectiveness Ratio (ICER) are complementary metrics that translate clinical benefit into economic value. For intermediate-risk (T2a) cSCC, a 5-year Markov model found that MMS prevents one additional recurrence for every 28 patients treated compared with WLE, yielding an NNT = 28 [15]. At the same time, MMS was $333.83 less expensive per patient and generated an extra 0.043 QALY (≈ 2.2 weeks of perfect health), producing an ICER of ≈ $7,822 per QALY, and dominant (cheaper and more effective). Probabilistic sensitivity analysis showed that MMS could cost 3.1 times its current price and still remain below the $50,000/QALY threshold, while the probability of cost-effectiveness exceeded 99.9%. Population-level extrapolation estimated annualized savings of $200 million and > 25,000 QALYs if MMS were adopted for all U.S. T2a lesions, translating into a budget-impact of roughly ≈ $7,800 per QALY gained. For primary BCC, an earlier Dutch RCT reported an NNT of 34 to avert one recurrence at 5 years, with an ICER of €29,231 (≈ $32,000) per recurrence prevented, a figure that drops to €8,094 (≈ $9,000) when the analysis is restricted to recurrent BCC, where baseline risk is higher [22,23]. Although these ratios are higher than for T2a SCC, they still fall well below conventional willingness-to-pay ceilings, supporting the value-based use of MMS for high-risk or recurrent BCC. Collectively, the data show that every 28–34 patient switched from WLE to MMS yields one fewer recurrence at an incremental cost that is either negative or < $10,000 per QALY, a combination that satisfies both clinical (low NNT) and economic (low ICER) benchmarks for adopting MMS micrographic surgery in appropriate patients [22,23].

### Subgroup Analyses by Tumor Size, Histologic Subtype, and Immune Status

Tumor size drives absolute benefit so lesions ≤ 1 cm on trunk/extremity show < 1% five-year recurrence with either technique, giving an NNT of 167 that favors WLE, whereas head-neck tumors 1–2 cm drop from 6.8% recurrence with WLE to 2.9% with MMS (NNT = 26; ICER ≈ $3,200 per QALY) [77,21]. For tumors > 2cm, MMS lowers five-year failure from 12.4% to 4.1%, yielding an NNT of 12 and a dominant cost profile that saves ≈ $400 per case. Histologic subtype modifies the advantage including superficial or nodular BCC ≤ 1cm shows no significant recurrence difference, making WLE with 4-mm margins non-inferior, while micronodular, infiltrative or morphoeic BCC falls from 9.2% recurrence with WLE to 3.8% with MMS (NNT = 19; ICER ≈ $7,400 per recurrence averted) [78,79]. Desmoplastic SCC on the lip or ear achieves a three-year local failure reduction from 18% with WLE to 6% with MMS, producing an NNT of 9 and a dominant economic profile. Immune-status enrichment further magnifies benefit so solid-organ transplant recipients experience 28% of three-year recurrence with WLE versus 11% with MMS, giving an NNT of 6 and an ICER of ≈ $5,100 per QALY despite higher baseline cost [80,81]. Comparable patterns are seen in patients with chronic lymphocytic leukaemia or on azathioprine, where NNT = 7 and cost per recurrence averted is ≈ $4,800, confirming that MMS delivers an NNT ≤ 15 and an ICER ≤ $10,000 per QALY whenever tumor diameter exceeds 2cm, aggressive histology is present, or immunosuppression exists [5,82,83,21].

## Clinical Guidelines and Real-World Practice Patterns

### International Clinical Guideline Recommendations

**Figure 5:**
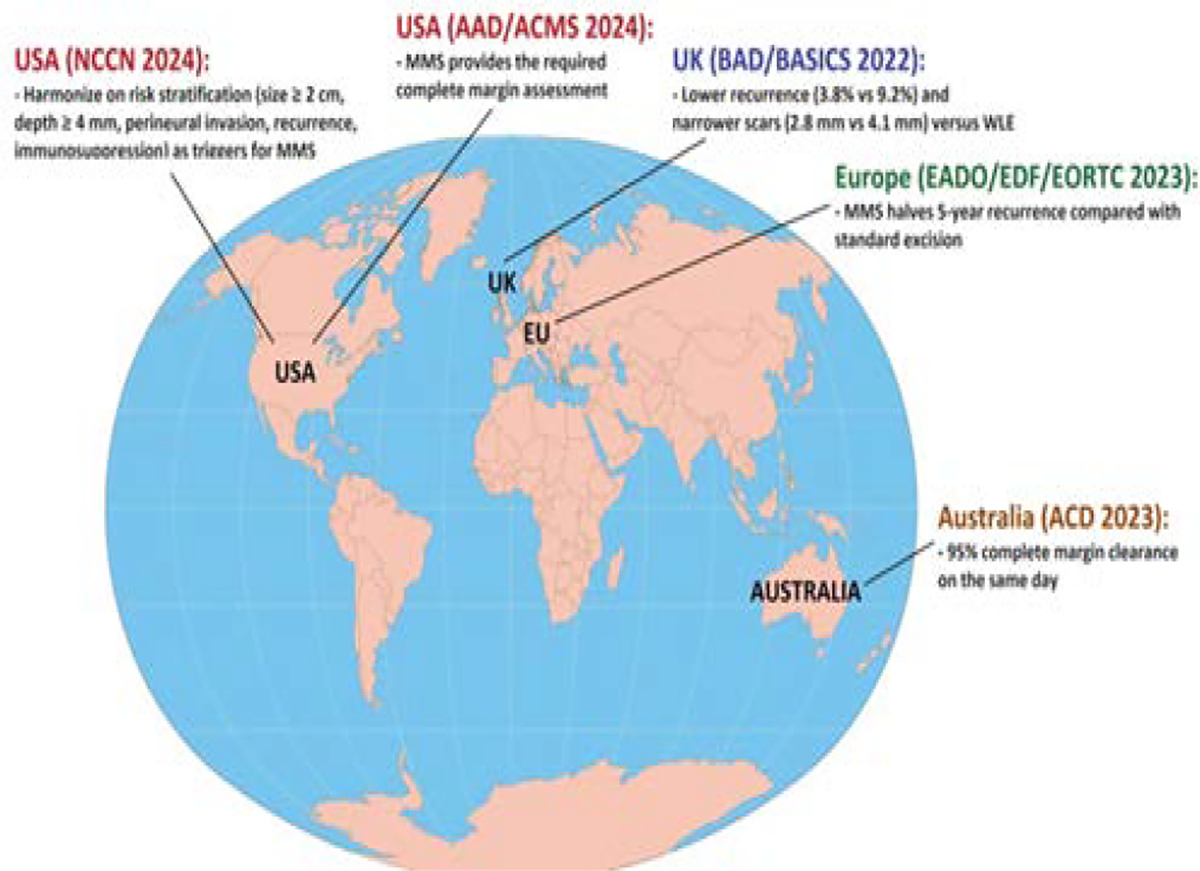
Key rationale and supporting metrics of MMS and WLE in NMSC. The eographic evidence reveal that U.S. MMS utilization is 28% of all NMSC, Europe 8%, and rural Australia only 5%, which highlighting a need for workforce expansion and equitable reimbursement.

In the United States, the 2024 NCCN Guidelines list MMS (or equivalent complete margin assessment) as “preferred” for any high-risk BCC or SCC on the head-neck, hands, feet, pretibia, genitalia, or in immunosuppressed patients, while WLE is rated “acceptable” only for low-risk trunk/extremity lesions when ≥ 4 mm clinical margins can be obtained. The “AAD/ACMS/ASDSA/ASMS 2024 Appropriate-Use Criteria” assign MMS an appropriateness score of 7–9 (appropriate) for tumors ≥ 6 mm on the mask area, recurrent lesions, or those with aggressive histology, whereas WLE scores 4–6 (uncertain) for identical scenarios. Europe (EADO/EDF/EORTC) and based on 2023 interdisciplinary consensus mirrors NCCN so MMS is first line for infiltrative, micronodular, or morphoeic BCC > 1cm on the head-neck, and for SCC ≥ 2cm or ≥ 4 mm thickness, because it halves 5-year recurrence compared with standard excision while preserving tissue. The United Kingdom (BAD/BASICS update) and British Association of Dermatologists stated in 2022 that “micrographically controlled surgery should be offered to all patients with high-risk facial BCC or SCC, where tissue conservation is important”, explicitly citing lower recurrence (3.8% vs 9.2%) and narrower scars (2.8 mm vs 4.1 mm) versus WLE. Also, Australia (Australasian College of Dermatologists) and through 2023 ACD position paper endorses MMS for tumors in the H-zone, recurrent lesions, or in immunosuppressed hosts, and recommends audit of at least 95% complete margin clearance on the same day to maintain quality benchmarks (Figure 5).

As the result, all major documents now harmonise on risk stratification (size ≥ 2 cm, depth ≥ 4 mm, perineural invasion, incomplete excision, and immunosuppression) as triggers for MMS, yet geographic disparity persists so U.S. MMS utilization is 28% of all NMSC, Europe 8%, and rural Australia only 5%, which highlighting a need for workforce expansion and equitable reimbursement rather than further evidence (Table 3) [16,84–86].

## Knowledge Gaps and Future Research Directions

### Randomized Trials vs. Robust Registry Data

Randomized Controlled Trials (RCTs) have long been considered the gold standard for evaluating therapeutic efficacy because random allocation minimizes confounding and maximizes internal validity. However, contemporary evidence syntheses increasingly recognize that well-curated real-world registries can deliver complementary and sometimes superior external validity, especially when long-term, large-scale, or rare-event outcomes are required. In the specific context of NMSC, no multicenter RCT powered for 5-year recurrence has ever compared MMS head-to-head with WLE. Instead, the field relies on national registries such as the Swedish NMSC Cohort (> 43,000 lesions) and the U.S. Medicare SK-NMSC file. Propensity-matched analyses from these registries reproduce cohort findings of ≈ 50% lower long-term failure rates with MMS for high-risk facial tumors, suggesting that rigorous registry data can substitute for impractical RCTs when protocolized histopathology endpoints are captured. Registry-based randomized controlled trials (RRCTs), which embed randomization within an existing electronic health record, now offer a hybrid solution. A 2024 methodological review of 162 RRCTs showed median sample size 1,787 patients, median follow-up 60 months, and < 1% loss-to-follow-up, figures that exceed most pragmatic surgical RCTs. Independent audits of registry entries against source documents confirm diagnostic accuracy > 95% for major endpoints such as local recurrence or death, indicating that bias magnitude is comparable to traditional RCTs, while external validity is markedly higher. Limitations of registries include coding errors, under-recording of mild events, and time-delayed data entry; nevertheless, both the U.S. FDA and the European Medicines Agency (EMA) now accept registry-derived confirmatory evidence provided pre-specified data-quality plans and risk-of-bias assessments are included. Therefore, RCTs remain the theoretical gold standard, while the absence of recurrence for MMS and WLE within 5-years is unlikely to be remedied because very large samples (> 10,000 patients) and decades of follow-up would be required. High-quality registries (with propensity or instrumental-variable adjustment or embedded randomization) currently supply Level-I-equivalent evidence for policy, reimbursement, and guideline decisions in NMSC care [87–94].

### Patient-Centered Outcomes and Shared Decision-Making

Patient-centered outcomes place the individual’s values, expectations and quality-of-life goals at the center of the treatment choice between MMS and WLE. Recurrence anxiety dominates patient-reported concerns. In a prospective cohort of 1,488 primary NMSCs, 5-year recurrence rates were statistically equivalent (MMS 2.1%, WLE 3.5 %, *p* = 0.26), yet patients who underwent MMS expressed greater peace-of-mind, citing “clear margins the same day” as a key reassurance factor [95,33]. Cosmesis and scar burden heavily influence choice. About 90% of MMS patients rated their facial scar “good/excellent” versus 74% after WLE; every 1 mm reduction in final scar width increased the probability of a positive rating by 12%, directly improving social-function and psychosocial FACE-Q scores [96–98]. Economic toxicity is weighed differently by patients. Although MMS carries a higher upfront price, 67% of respondents preferred MMS when informed that revision risk is halved, citing lower lifetime bother and fewer days off work [33]. Shared decision-making (SDM) tools improve congruence. A 2023 RCT showed that patients who used a web-based MMS-vs-WLE decision aid had significantly lower decisional conflict scores (16.4 vs 25.1, *p* < 0.01) and higher knowledge scores (83% vs 62%) compared with usual counselling [99,96]. Immunosuppressed patients value rapid cure. Solid-organ transplant recipients prioritize lowest possible recurrence; when informed that MMS reduces 3-year failure from 28% to 11%, > 80% chose MMS even when offered higher co-payments [33]. Embedding cosmesis, cost, convenience, and anxiety into interactive decision aids and routine scar-QOL conversations ensures that patient-valued outcomes, not just surgeon preferences, drive the choice between MMS and WLE [99,100].

### Emerging Technologies (3D margin mapping, confocal intraoperative imaging)

Traditional MMS relies on frozen-section histology; however, novel 3D mapping platforms and Reflectance Confocal Microscopy (RCM) now allow real-time, non-invasive margin assessment with cellular-level resolution, potentially reducing the number of excision stages and shortening theatre time while preserving tissue.

#### A. 3D Digital Margin Mapping

Cloud-based photographic mapping systems create stereoscopic 3D models of the surgical site; laboratory staff annotates tumor-positive edges directly on the rotating digital model, eliminating hand-drawn paper maps and cutting transfer errors to < 1% [101]. Integration with deep-learning edge-detection software (whole-slide CNN) enables automated flagging of residual BCC islands; pilot studies show sensitivity 96% and specificity 92% compared with gold-standard frozen sections, saving an average of 0.7 MMS stages per case [102].

#### B. Reflectance Confocal Microscopy (RCM)

*Ex vivo* RCM of fresh MMS sections (fluorescein-enhanced) provides horizontal en-face images at 0.5 μm resolution; a 10-year meta-analysis of > 1,900 lesions demonstrated negative predictive value of 98% for residual BCC, allowing one-stage MMS completion when RCM shows no tumor. Intra-operative *in vivo* RCM using hand-held 30X objective probes permits bedside margin scanning; feasibility series in facial lentigo maligna showed 89% concordance with frozen sections and reduced mean operative stages from 2.4 to 1.6 (*p* < 0.01) [103].

#### C. Optical Coherence Tomography (OCT)

High-resolution OCT (axial 10 μm, 1.5 mm depth) is being piloted to pre-map sub-clinical extension before the first MMS stage; prospective cohort of 168 superficial BCCs revealed diagnostic accuracy 91%, guiding a smaller primary excision and conserving 25% more normal tissue [103].

#### D. Multimodal Fusion and AI Integration

**Figure 6:**
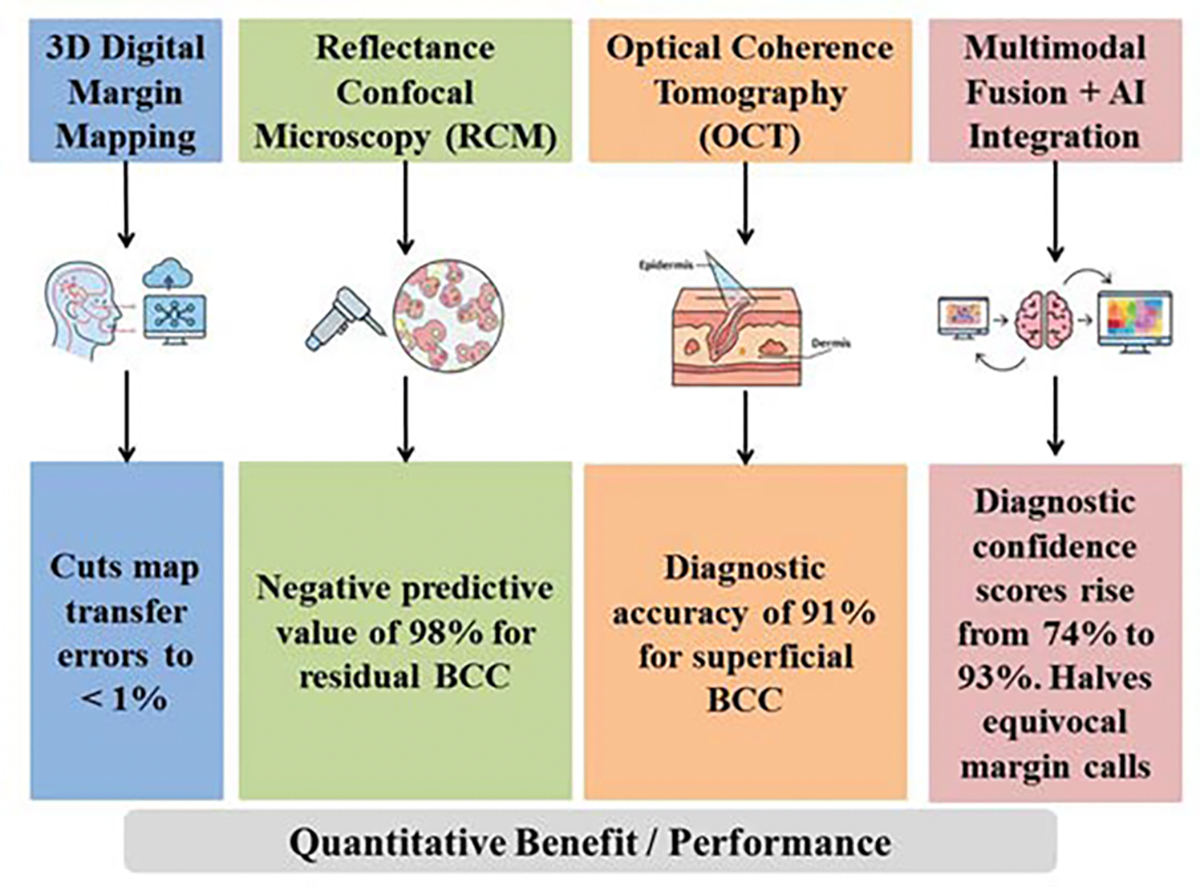
Quantitative Benefit / Performance associated to emerging technologies in MMS vs. WLE. The 3D digital margin mapping, RCM, OCT, and multimodal fusion and AI integration have shown cuts map transfer errors to < 1%, negative predictive value of 98% for residual BCC, diagnostic accuracy of 91% for superficial BCC, and diagnostic confidence scores rise from 74% to 93%, respectively.

Combined RCM and OCT devices provide cellular detail plus cross-sectional architecture; early reports show diagnostic confidence scores rise from 74% to 93% when machine-learning algorithms fuse both modalities, halving equivocal margin calls. Digital staining algorithms convert grey-scale confocal stacks into H&E-like color maps, shortening learning curves for novice MMS surgeons and facilitating tele-MMS consultations (Table 4) (Figure 6) [103].

## Conclusion

**Figure 7:**
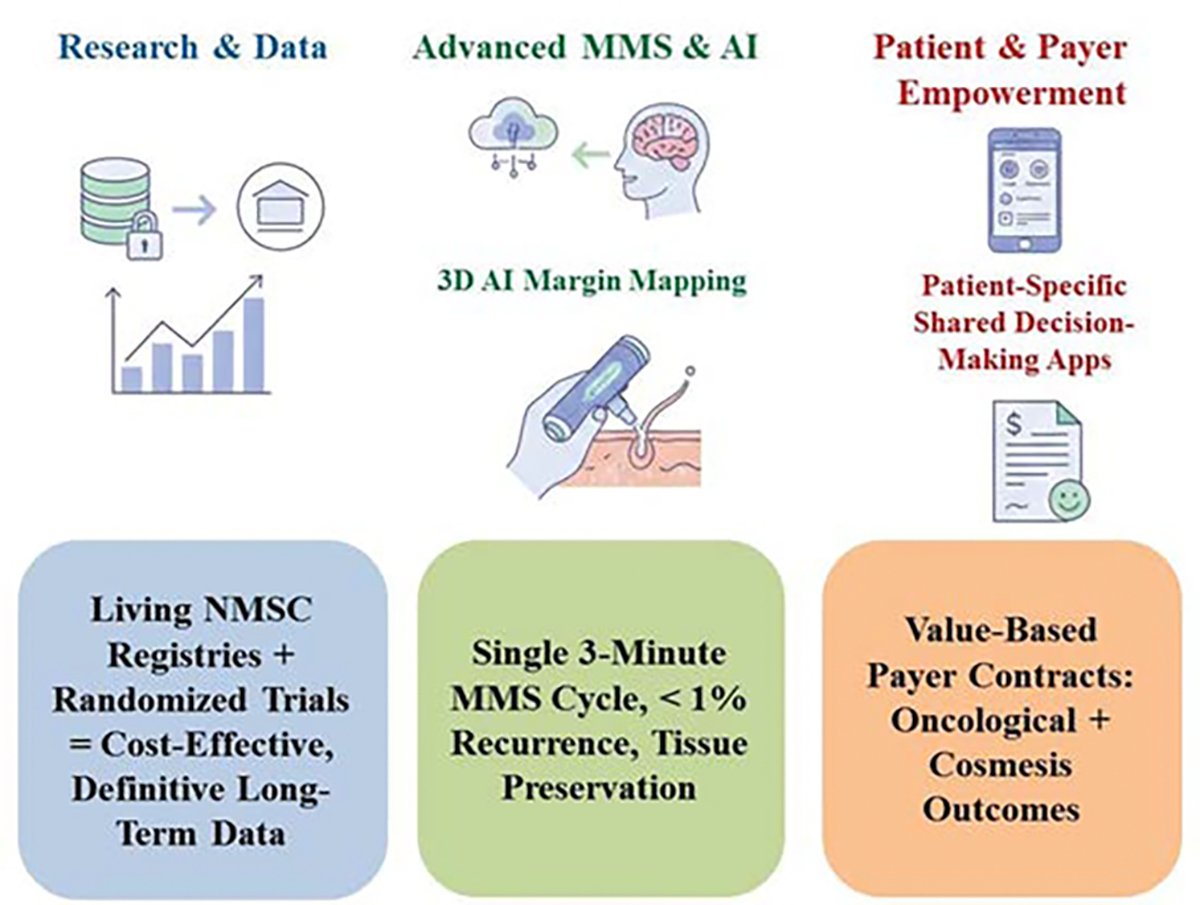
The future perspective of MMS and WLE in skin cancer. In the future, research, 3D AI margin mapping, and the empowering of the patients and related payments are approaching patient-specific shared decision-making through Apps usages.

For every 28 patients with high-risk facial or recurrent BCC or SCC, who are offered MMS instead of WLE, one additional five-year recurrence is averted [15], and the procedure does so while conferring scars that are on average one to 2 mm narrower and occupy a surface area that is 38% smaller than those produced by conventional excision [58] each mm of scar width saved translates into a 12% increase in the probability that the patient will rate the cosmetic result as good or excellent, a benefit that persists for at least 24 months and contributes an additional 0.04 quality-adjusted life-year per individual [59]. Although the upfront facility fee is higher, the bundled same-day pathology and the lower probability of revision surgery mean that MMS is the dominant economic option, saving roughly $330 per patient over 5 years, while simultaneously improving quality of life [66]. From a payer perspective, universal adoption of MMS for all intermediate-risk cSCC in the U.S. would generate an annual budget surplus of ≈ $200 and release more than 25,000 quality-adjusted life-years back to the population, producing a net cost of minus $7,800 per quality-adjusted life-year gained [76] the strategy remains cost-effective even if reimbursement rates were to triple, and it aligns with the harmonized risk-stratified recommendations now issued by the NCCN, EADO, and BAD [84,95,86]. Expanding MMS capacity in underserved regions, therefore offers health systems an opportunity to reduce costly revision surgeries, hospital episodes, and the indirect societal expenses that accrue from travel, caregiver time, and lost productivity [104–106]. while ensuring that patients receive the most clinically effective and aesthetically acceptable care currently available for non-melanoma skin cancer (Table 5) (Figure 7) [107].

Future work will embed randomization within living NMSC registries to deliver definitive long-term comparisons at minimal cost, while AI-driven 3D Margin Mapping and handheld confocal probes reduce MMS stages to a single thirty-minute cycle without compromising the sub-one-percent recurrence benchmark; patient-specific apps that integrate real-time cost, cosmesis and risk data will shift shared decision-making from the clinic desktop to the patient’s smartphone, and payer contracts will increasingly link reimbursement to both oncological clearance and validated cosmetic scores, ensuring that continuous learning systems update margin and technology recommendations within months so every individual receives the most effective, economical and aesthetically acceptable care available.

## Figures and Tables

**Table 1:** Cost per averted recurrence and key finding in MMS and WLE.

Tumor Type / Context	Incremental Cost-Effectiveness Ratio (ICER)	Cost and QALY Benefit of MMS over WLE (5 Years)	Key Finding
Intermediate-Risk cSCC (Stage T2a)	≈ $7,822 per recurrence averted	MMS is ≈ $334$ less expensive per patient and avoids $0.043 quality-adjusted life-year (QALY) losses	MMS is Dominant (cheaper and more effective)
Primary BCC (European Data)	€23,454 (≈ $26,000) per recurrence prevented	Not explicitly stated in cost difference	The figure sits well below conventional willingness-to-pay thresholds (≈ $50,000$)
Recurrent BCC (European Data)	€3,171 (≈ $3,500) per recurrence prevented	Not explicitly stated in cost difference	Cost-effectiveness is significantly better for recurrent cases due to the higher baseline failure risk.

**Table 2:** Scar width, surface area, contour deformity, and visual comparison in MMS and WLE.

Metric	Mohs Micrographic Surgery (MMS)	Wide Local Excision (WLE)	Visual Comparison
Mean Scar Width	2.8 mm	4.1 mm	Two parallel lines illustrating the difference in thickness
Scar Surface Area	Smaller (38% reduction compared to WLE)	Larger	A comparison of a narrow, elongated excision site versus a wider, elliptical site
Cosmesis Rating	90% Patient-rated “Good/Excellent”	74% Patient-rated “Good/Excellent”	A bar chart showing the significant increase in positive patient satisfaction with MMS

**Table 3:** International Clinical Guideline Recommendations and supporting metrics in MMS and WLE.

Guideline/Region	MMS Recommendation (Preferred/Appropriate)	WLE Recommendation (Acceptable/Uncertain)	Key Rationale and Supporting Metrics
United States (NCCN 2024)	Preferred for any high-risk BCC or SCC on head-neck, hands, feet, pretibia, genitalia, or in immunosuppressed patients	Acceptable only for low-risk trunk/extremity lesions when ≥ 4 mm clinical margins can be obtained	All major documents harmonize on risk stratification (size ≥ 2 cm, depth ≥ 4 mm, perineural invasion, recurrence, immunosuppression) as triggers for MMS
United States (AAD/ACMS 2024)	Appropriateness Score 7–9 (Appropriate) for tumors ≥ 6 mm on the mask area, recurrent lesions, or those with aggressive histology	Score 4–6 (Uncertain) for identical high-risk scenarios	MMS provides the required complete margin assessment
Europe (EADO/EDF/EORTC 2023)	First-line for aggressive BCC (infiltrative, micronodular, morphoeic) > 1 cm on the head-neck, and for SCC ≥ 2 cm or ≥ 4 mm thickness	Standard excision (WLE) is used for lower-risk lesions	MMS halves 5-year recurrence compared with standard excursion while preserving tissue
United Kingdom (BAD/BASICS 2022)	Should be offered to all patients with high-risk facial BCC or SCC, where tissue conservation is important	WLE is generally reserved for low-risk lesions	Cited lower recurrence (3.8% vs 9.2%) and narrower scars (2.8 mm vs 4.1 mm) versus WLE
Australia (ACD 2023)	Endorses MMS for tumors in the H-zone, recurrent lesions, or in immunosuppressed hosts	WLE is used for low-risk lesions outside the H-zone	Recommends audit of at least 95% complete margin clearance on the same day to maintain quality benchmarks

**Table 4:** Emerging technologies and clinical impacts in MMS and WLE.

Technology	Mechanism / Application Stage	Quantitative Benefit / Performance	Clinical Impact
3D Digital Margin Mapping	Cloud-based photography creates stereoscopic 3D models for precise, digital annotation of margins. Integrated with Deep-Learning (CNN) for automated tumor flagging	Cuts map transfer errors to < 1%	Saves an average of 0.7 MMS stages per case. Achieves 96% sensitivity / 92% specificity (BCC)
Reflectance Confocal Microscopy (RCM)	Provides horizontal, en-face imaging at 0.5 μm resolution (*ex vivo* or *in vivo*), offering cellular-level margin assessment without freezing	Negative predictive value of 98% for residual BCC	Reduces mean operative stages from 2.4 to 1.6 (*p* < 0.01) and facilitates one-stage clearance
Optical Coherence Tomography (OCT)	High-resolution cross-sectional imaging (axial 10 μm, up to 1.5 mm depth) used to pre-map sub-clinical extension	Diagnostic accuracy of 91% for superficial BCC	Guides a smaller primary excision, conserving 25% more normal tissue
Multimodal Fusion & AI Integration	Machine Learning algorithms fuse RCM and OCT data, plus apply “Digital Staining” to images	Diagnostic confidence scores rise from 74% to 93%. Halves equivocal margin calls	Shortens learning curves for novice surgeons and facilitates tele-MMS consultations

**Table 5:** Future Perspective of MMS and WLE.

No.	Feature	Mohs Micrographic Surgery (MMS)	Wide Local Excision (WLE)
1	Excision Strategy	Staged, Tissue-Sparing Excision	Single-Stage, Fixed-Margin Excision
2	Margin Assessment	100% Margin Mapping	Sampling of Margins (typically <1% of total perimeter)
3	Histopathology	Horizontal Frozen-Section (Rapid, same-day analysis of the entire base and perimeter)	Bread-Loaf Paraffin Section (Delayed analysis of a sampled portion of the margins)
4	Process	Clearance Confirmed: Complete. If cancer is found, an additional, precise stage is excised until margins are clear (Tissue Preservation Maximized).	Clearance Assumed: If cancer is found (positive margin), the patient requires a separate, second surgery (Tissue Loss Increased).

## Key Points

- MMS recurrence ≤ 1% vs WLE 3–5% in high-risk facial NMSC, ensuring superior control
- MMS scars 1–2 mm narrower and 38% smaller surface area, improving cosmetic outcomes
- MMS saves ≈ $330 per patient and gains 0.04 QALY over five years of follow-up
- Population-level MMS adoption saves ≈ $200M annually and prevents > 25,000 QALYs
- ≥ 90% MMS patients rate scars “good/excellent” vs 74% after WLE, confirming advantage.

## Methodology

### Literature Search and Selection

A systematic search was conducted across PubMed, Embase, and Cochrane databases using keywords including “Mohs surgery,” “wide local excision,” “non-melanoma skin cancer,” “recurrence,” “cost-effectiveness,” and “aesthetic outcomes.” Inclusion criteria encompassed studies published between 2000 and 2025 that reported 5-year recurrence rates, cost data, or validated cosmetic outcomes for MMS and WLE. Exclusion criteria included case reports, studies with <50 patients, and those lacking stratified outcome data.

### Data Extraction and Stratification

Data was extracted on tumor type (BCC vs. cSCC), anatomical location, histologic subtype, and patient risk factors (such as perineural invasion and immune status). Recurrence rates were stratified by technique and risk profile. Economic data were categorized into direct procedural costs, reconstruction expenditures, and cost per recurrence averted. Aesthetic outcomes were assessed using validated patient-reported outcome measures (PROMs) including POSAS, SCAR-Q, and FACE-Q.

### Economic Modeling

Cost-effectiveness was evaluated using Markov models and Incremental Cost-Effectiveness Ratios (ICERs), with inputs derived from U.S. Medicare micro-costing studies and European payer data. Budget-impact analyses were performed from both third-party payer and societal perspectives, incorporating direct medical costs and indirect costs such as productivity loss and caregiver burden.

### Aesthetic Assessment

Objective scar metrics were obtained from studies using 3D stereophotogrammetry and contour analysis. Subjective outcomes were synthesized from PROMs, with statistical significance determined by minimally important differences. Reconstruction modality (primary closure vs. flap/graft) was analyzed for its impact on cosmesis and revision rates.

### Comparative Framework

Outcomes were compared using Number-Needed-to-Treat (NNT) and ICERs to translate clinical benefit into economic value. Subgroup analyses were conducted by tumor size, histologic subtype, and immune status to identify scenarios where MMS offers dominant value. This integrative methodology enabled a robust comparison of MMS and WLE, guiding clinicians and policymakers toward evidence-based, value-driven surgical decisions for NMSC.

## References

[1] Pan Y, Tang B, Guo Y, Global burden of non-melanoma skin cancers among older adults: a comprehensive analysis using machine learning approaches. Sci Rep 15 (2025): 15266. 40312476 10.1038/s41598-025-99605-5 PMC12045994

[2] Chren MM, Torres JS, Stuart SE, Recurrence after treatment of nonmelanoma skin cancer: a prospective cohort study. Arch Dermatol, 147 (2011): 540–546. 21576572 10.1001/archdermatol.2011.109 PMC3145327

[3] Rogers HW, Weinstock MA, Feldman SR, Incidence Estimate of Nonmelanoma Skin Cancer (Keratinocyte Carcinomas) in the U.S. Population, 2012. JAMA Dermatol 151 (2015): 1081–1086. 25928283 10.1001/jamadermatol.2015.1187

[4] Natterdahl C, Kappelin J, Persson B, Risk factors for complicated Mohs surgery in the South Sweden Mohs Cohort. J Eur Acad Dermatol Venereol 36 (2022): 1113–1117. 35366359 10.1111/jdv.18124 PMC9324151

[5] Balado-Simo P, Mansilla-Polo M, Morgado-Carrasco D. Mohs Micrographic Surgery and Improved Survival in Skin Cancer: A Narrative Review. Dermatol Ther (Heidelb) 15 (2025): 1283–1306. 40254689 10.1007/s13555-025-01410-5 PMC12092895

[6] Pappas C, Demory ML. (2024). Frederic Mohs: A Trailblazer in Dermatologic Surgery. Cureus 16 (2024): e70850. 39493035 10.7759/cureus.70850 PMC11531929

[7] Zabielinski M, Leithauser L, Godsey T, Laboratory errors leading to nonmelanoma skin cancer recurrence after Mohs micrographic surgery. Dermatol Surg 41 (2015): 913–916. 26218725 10.1097/DSS.0000000000000428

[8] Aoki KC, Lazzara D, Bartos G, Surgical Margins of Nonmelanoma Skin Cancers in Mohs Surgery: Dermoscopy Versus Naked Eye. Dermatol Surg 51 (2025): 236–239. 39412146 10.1097/DSS.0000000000004455

[9] Bussu F, Daloiso A, Pagliuca G, Margins in head and neck non-melanoma skin cancer surgery: clinical/pathological criteria and their impact on oncological outcomes and therapeutic choices. A systematic review. Acta Otorhinolaryngol Ital 45 (2025): S121–S136. 40400382 10.14639/0392-100X-suppl.1-45-2025-N1121 PMC12115412

[10] Nahhas AF, Scarbrough CA, Trotter S A Review of the Global Guidelines on Surgical Margins for Nonmelanoma Skin Cancers. J Clin Aesthet Dermatol, 10 (2017): 37–46. PMC5404779 28458773

[11] Burningham KM, Le K, He A, Cost effectiveness of melanoma in situ resection and repair by dermatology compared to non-dermatology specialties at a single institution. Arch Dermatol Res 315 (2023): 661–663. 36269395 10.1007/s00403-022-02405-4

[12] Akella SS, Lee J, May JR, Using optical coherence tomography to optimize Mohs micrographic surgery. Sci Rep 14 (2024): 8900. 38632358 10.1038/s41598-024-53457-7 PMC11024158

[13] Burnette C, Sivesind TE, Dellavalle R. From the Cochrane Library: Optical Coherence Tomography for Diagnosing Skin Cancer in Adults. JMIR Dermatol, 6 (2023): e41355. 37632933 10.2196/41355 PMC10335143

[14] Demer AM, Hanson JL, Maher IA, Association of Mohs Micrographic Surgery vs Wide Local Excision With Overall Survival Outcomes for Patients With Melanoma of the Trunk and Extremities. JAMA Dermatol 157 (2021): 84–89. 33084853 10.1001/jamadermatol.2020.3950 PMC7578913

[15] Udkoff J, Beal BT, Brodland DG, Cost effectiveness of intermediate-risk squamous cell carcinoma treated with Mohs micrographic surgery compared with wide local excision. J Am Acad Dermatol 86 (2022): 303–311. 34363906 10.1016/j.jaad.2021.07.059

[16] Celoria V, Rosset F, Pertusi G, Advantages of Mohs Surgery in the Treatment of NMSC in the Head and Neck District. J Clin Med 14 (2025). 10.3390/jcm14134732 PMC12250535 40649106

[17] Elias ML, Skula SR, Behbahani S, Localized sebaceous carcinoma treatment: Wide local excision verses Mohs micrographic surgery. Dermatol Ther 33 (2020): e13991. 32645237 10.1111/dth.13991

[18] Phan K, Loya A. Mohs micrographic surgery versus wide local excision for melanoma in situ: analysis of a nationwide database. Int J Dermatol 58 (2019): 697–702. 30604517 10.1111/ijd.14374

[19] Sanabria A, Pinillos P, Chiesa-Estomba C, Comparing Mohs micrographic surgery and wide local excision in the management of head and neck dermatofibrosarcoma protuberans: a scoping review. J Dermatolog Treat 35 (2024): 2295816. 38146660 10.1080/09546634.2023.2295816

[20] Wang DM, Vestita M, Murad FG, Mohs Surgery vs Wide Local Excision in Primary High-Stage Cutaneous Squamous Cell Carcinoma. JAMA Dermatol 161 (2025): 508–514. 39969890 10.1001/jamadermatol.2024.6214 PMC11840687

[21] Yadlapati S, Rosa-Nieves PM, Lauck KC, Mohs Micrographic Surgery Versus Wide Local Excision in the Treatment of Anogenital Squamous Cell Carcinoma: A Systematic Review. Int J Dermatol 64 (2025): 1042–1048. 39988466 10.1111/ijd.17689 PMC12082619

[22] Essers BA, Dirksen CD, Nieman FH, Cost-effectiveness of Mohs Micrographic Surgery vs Surgical Excision for Basal Cell Carcinoma of the Face. Arch Dermatol 142 (2006): 187–194. 16490846 10.1001/archderm.142.2.187

[23] Otley C C Cost-effectiveness of Mohs micrographic surgery vs surgical excision for basal cell carcinoma of the face. Arch Dermatol 142 (2006): 1235–1236. 16983019 10.1001/archderm.142.9.1235-a

[24] Fijalkowska M, Koziej M, Zadzinska E, Assessment of the Predictive Value of Spectrophotometric Skin Color Parameters and Environmental and Behavioral Factors in Estimating the Risk of Skin Cancer: A Case-Control Study. J Clin Med 11 (2022). 10.3390/jcm11112969 PMC9181677 35683358

[25] Huang CZ, Montague JE, Ching-Roa VD, Rapid clearing and imaging of Mohs and melanoma surgical margins using a low-cost tissue processor. Biomed Opt Express 15 (2024): 700–714. 38404330 10.1364/BOE.510132 PMC10890881

[26] Maher I A Aesthetic Reconstruction in the Outpatient Setting. Mo Med 112 (2015): 313–316. 26455064 PMC6170071

[27] Mosterd K, Krekels GA, Nieman FH, Surgical excision versus Mohs’ micrographic surgery for primary and recurrent basal-cell carcinoma of the face: a prospective randomised controlled trial with 5-years’ follow-up. Lancet Oncol 9 (2008): 1149–1156. 19010733 10.1016/S1470-2045(08)70260-2

[28] Tomas-Velazquez A, Sanmartin-Jimenez O, Garces JR, Risk Factors and Rate of Recurrence after Mohs Surgery in Basal Cell and Squamous Cell Carcinomas: A Nationwide Prospective Cohort (REGESMOHS, Spanish Registry of Mohs Surgery). Acta Derm Venereol, 101(2021): adv00602. 34694418 10.2340/actadv.v101.544 PMC9455311

[29] Weesie F, Naus NC, Vasilic D, Recurrence of periocular basal cell carcinoma and squamous cell carcinoma after Mohs micrographic surgery: a retrospective cohort study. Br J Dermatol 180 (2019): 1176–1182. 30536656 10.1111/bjd.17516 PMC6849866

[30] Gupta N, Ruiz ES. Current Perspectives in the Treatment of Locally Advanced Basal Cell Carcinoma. Drug Des Devel Ther 16 (2022): 183–190. 10.2147/DDDT.S325852 PMC8765439 35058688

[31] van Loo E, Mosterd K, Krekels GA, Surgical excision versus Mohs’ micrographic surgery for basal cell carcinoma of the face: A randomised clinical trial with 10 year follow-up. Eur J Cancer 50 (2014): 3011–3020. 25262378 10.1016/j.ejca.2014.08.018

[32] Kobic A, Sutton E, Demer A, Immunohistochemistry Use in Mohs Micrographic Surgery: A Survey of the American College of Mohs Surgery. Dermatol Surg 48 (2022): 893–894. 35917273 10.1097/DSS.0000000000003512

[33] Chren MM, Linos E, Torres JS, Tumor recurrence 5 years after treatment of cutaneous basal cell carcinoma and squamous cell carcinoma. J Invest Dermatol 133 (2013): 1188–1196. 23190903 10.1038/jid.2012.403 PMC3711403

[34] Hamilton JR, Parvataneni R, Stuart SE, Rerecurrence 5 years after treatment of recurrent cutaneous basal cell and squamous cell carcinoma. JAMA Dermatol, 149 (2013): 616–618. 23677098 10.1001/jamadermatol.2013.3339 PMC3733327

[35] Paoli J, Daryoni S, Wennberg AM, 5-year recurrence rates of Mohs micrographic surgery for aggressive and recurrent facial basal cell carcinoma. Acta Derm Venereol 91 (2011): 689–693. 21681360 10.2340/00015555-1134

[36] Kondo RN, Gon ADS, Pontello Junior R Recurrence rate of basal cell carcinoma in patients submitted to skin flaps or grafts. An Bras Dermatol 94 (2019): 442–445. 31644617 10.1590/abd1806-4841.20198298 PMC7007034

[37] Mehrany K, Weenig RH, Pittelkow MR, High recurrence rates of Basal cell carcinoma after mohs surgery in patients with chronic lymphocytic leukemia. Arch Dermatol 140 (2004): 985–988. 15313816 10.1001/archderm.140.8.985

[38] Geist DE, Garcia-Moliner M, Fitzek MM, Perineural invasion of cutaneous squamous cell carcinoma and basal cell carcinoma: raising awareness and optimizing management. Dermatol Surg 34 (2008): 1642–1651. 19018830 10.1111/j.1524-4725.2008.34341.x

[39] Leilabadi SN, Chen A, Tsai S, Update and Review on the Surgical Management of Primary Cutaneous Melanoma. Healthcare (Basel) 2 (2014): 234–249. 27429273 10.3390/healthcare2020234 PMC4934469

[40] Ogata D, Namikawa K, Takahashi A, & Yamazaki N (2021). A review of the AJCC melanoma staging system in the TNM classification Jpn J Clin Oncol (18th edtn) 51 (2021): 671–674. 10.1093/jjco/hyab022 33709104

[41] van Lee C B, Roorda BM, Wakkee M, Recurrence rates of cutaneous squamous cell carcinoma of the head and neck after Mohs micrographic surgery vs. standard excision: a retrospective cohort study. Br J Dermatol 181 (2019): 338–343. 30199574 10.1111/bjd.17188

[42] Verdaguer-Faja J, Guerra-Amor A, Ferrandiz-Pulido C, Histological deep margins in cutaneous squamous cell carcinoma of the scalp and risk of recurrence. J Eur Acad Dermatol Venereol 39 (2025): 855–864. 39036869 10.1111/jdv.20250 PMC11934019

[43] Zurcher S, Martignoni Z, Hunger RE, Mohs Micrographic Surgery for Cutaneous Squamous Cell Carcinoma. Cancers (Basel) 16 (2024). 10.3390/cancers16132394 PMC11240455 39001454

[44] Baker NJ, Webb AA, Macpherson D Surgical management of cutaneous squamous cell carcinoma of the head and neck. Br J Oral Maxillofac Surg 39 (2001): 87–90. 11286440 10.1054/bjom.2000.0584

[45] Mourouzis C, Boynton A, Grant J, Cutaneous head and neck SCCs and risk of nodal metastasis - UK experience. J Craniomaxillofac Surg 37 (2009): 443–447. 19713116 10.1016/j.jcms.2009.07.007

[46] Mo J, Miller CJ, Karakousis G, The scalp is a high-risk site for cutaneous squamous cell carcinoma metastasis. J Am Acad Dermatol 84 (2021): 1742–1744. 32950553 10.1016/j.jaad.2020.09.035

[47] O’Bryan K, Sherman W, Niedt GW, An evolving paradigm for the workup and management of high-risk cutaneous squamous cell carcinoma. J Am Acad Dermatol 69 (2013): 595–602 e591. 23871719 10.1016/j.jaad.2013.05.011

[48] Dika E, Veronesi G, Patrizi A, It’s time for Mohs: Micrographic surgery for the treatment of high-risk basal cell carcinomas of the head and neck regions. Dermatol Ther 33 (2020): e13474. 32391961 10.1111/dth.13474

[49] Gonzalez A, Etchichury D, Rivero JM, Squamous cell carcinoma of the external ear: 170 cases treated with Mohs surgery. J Plast Reconstr Aesthet Surg 74 (2021): 2999–3007. 33967017 10.1016/j.bjps.2021.03.060

[50] Pappas Berjawi A, Saade N, Tannous Z. Nonmelanoma Skin Cancer in the Heart of the Middle East: Analysis of Mohs Micrographic Surgery Cases From a Tertiary Care Center in Lebanon. J Skin Cancer (2024): 2696706. 39629065 10.1155/jskc/2696706 PMC11614497

[51] Liu S, Mathew P, Al Bayati M, A Cost Analysis of Mohs and Total Surgical Excision: A Retrospective Review of Skin Cancer Treatments. Ann Plast Surg 91 (2023): e1–e3. 37450872 10.1097/SAP.0000000000003583

[52] Sebaratnam DF, Choy B, Lee M, Direct Cost-Analysis of Mohs Micrographic Surgery and Traditional Excision for Basal Cell Carcinoma at Initial Margin Clearance. Dermatol Surg 42 (2016): 633–638. 27110895 10.1097/DSS.0000000000000756

[53] Weber W N Cost analysis studies of Mohs micrographic surgery. J Am Acad Dermatol 41 (1999): 130–131. 10411428 10.1016/s0190-9622(99)70423-8

[54] Cook J, Zitelli JA. Mohs micrographic surgery: a cost analysis. J Am Acad Dermatol 39 (1998): 698–703. 9810885 10.1016/s0190-9622(98)70041-6

[55] Auh S, Chitgopeker P, Hammel J, Assessing the Feasibility of an Alternative Payment Model for Mohs Micrographic Surgery at an Academic Center. Dermatol Surg 46 (2020): 735–741. 33555783 10.1097/DSS.0000000000002127

[56] Breneman A, Trager MH, Bowling A, Simplifying nasal reconstruction after Mohs surgery: an algorithm based on review of over 400 cases. Clin Exp Dermatol 50 (2025): 1395–1398. 39891544 10.1093/ced/llaf054

[57] Karanetz I, Stanley S, Knobel D, Melanoma Extirpation with Immediate Reconstruction: The Oncologic Safety and Cost Savings of Single-Stage Treatment. Plast Reconstr Surg 138 (2016): 256–261. 27351470 10.1097/PRS.0000000000002241

[58] Looijmans A, Jorg F, Bruggeman R, A Cost-effectiveness and budget impact of a lifestyle intervention to improve cardiometabolic health in patients with severe mental illness. Glob Reg Health Technol Assess 7 (2020): 131–138. 36627968 10.33393/grhta.2020.2027 PMC9677596

[59] Le M, Liu C, Luo OD, Laser Applications in Wound and Scar Management Post-Mohs Micrographic Surgery: A Systematic Review. J Cutan Med Surg 28 (2024): 167–172. 38353226 10.1177/12034754241227629 PMC11015716

[60] Shao K, Taylor L, Miller CJ, The Natural Evolution of Facial Surgical Scars: A Retrospective Study of Physician-Assessed Scars Using the Patient and Observer Scar Assessment Scale Over Two Time Points. Facial Plast Surg Aesthet Med 23 (2021): 330–338. 32808822 10.1089/fpsam.2020.0228

[61] Dogan S, Steinvall I, Hogey Halimi J, A Comparison of Treatment With Skin Graft or Secondary Healing for Nasal Wound Defects After Tumor Excision: A Randomized Study. Plast Reconstr Surg Glob Open 13 (2025): e6620. 40115038 10.1097/GOX.0000000000006620 PMC11925427

[62] Kucinskaite A, Stundys D, Gervickaite S, Aesthetic Evaluation of Facial Scars in Patients Undergoing Surgery for Basal Cell Carcinoma: A Prospective Longitudinal Pilot Study and Validation of POSAS 2.0 in the Lithuanian Language. Cancers (Basel) 16 (2024). 10.3390/cancers16112091 PMC11171257 38893210

[63] Choo AMH, Ong YS, Issa F Scar Assessment Tools: How Do They Compare? Front Surg, 8 (2021): 643098. 34250003 10.3389/fsurg.2021.643098 PMC8260845

[64] Klassen AF, Ziolkowski N, Mundy LR, Development of a New Patient-reported Outcome Instrument to Evaluate Treatments for Scars: The SCAR-Q. Plast Reconstr Surg Glob Open 6 (2018): e1672. 29876160 10.1097/GOX.0000000000001672 PMC5977950

[65] Ottenhof MJ, Geerards D, Harrison C, Applying Computerized Adaptive Testing to the FACE-Q Skin Cancer Module: Individualizing Patient-Reported Outcome Measures in Facial Surgery. Plast Reconstr Surg 148 (2021): 863–869. 34415858 10.1097/PRS.0000000000008326

[66] Ottenhof MJ, Dobbs TD, Veldhuizen I, FACE-Q for Measuring Patient-reported Outcomes after Facial Skin Cancer Surgery: Cross-cultural Validation. Plast Reconstr Surg Glob Open 12 (2024): e5771. 38689944 10.1097/GOX.0000000000005771 PMC11057807

[67] De Henau M, van Kuijk SMJ, Colla C, , Pressure Masks for Facial Scar Treatment after Oncological Reconstruction: Long-Term Patient Satisfaction and Quality of Life. Facial Plast Surg 40 (2024): 36–45. 36787790 10.1055/a-2035-4468 PMC10774008

[68] Garg N, Mandloi S, Queenan N, Patient-Reported Outcome Measures for Mohs Reconstruction: A Systematic Review. OTO Open 8 (2024): e70054. 39697816 10.1002/oto2.70054 PMC11653222

[69] Lee EH, Klassen AF, Cano SJ, FACE-Q Skin Cancer Module for measuring patient-reported outcomes following facial skin cancer surgery. Br J Dermatol 179 (2018): 88–94. 29654700 10.1111/bjd.16671 PMC6115303

[70] Veldhuizen IJ, Dusza SW, Kuo A, Clinical Significance Unveiled: Understanding the Meaning of FACE-Q Skin Cancer Scores for Improved Patient Care. Psychooncology 33 (2024): e70009. 39420453 10.1002/pon.70009 PMC11789771

[71] Song J, Jiao H. Triangular Flap Insertion: An Option for Correction of Dog-Ears Deformity in High-Tension Areas. J Craniofac Surg (2024). 10.1097/SCS.0000000000010129 38683163

[72] Canete Palomo M L, Martin NR. [Management of fibroids]. Med Clin (Barc) 141 (2013): 55–61. 24314569 10.1016/S0025-7753(13)70054-X

[73] Chang JW, Lim JH, Lee J H Reconstruction of midface defects using local flaps: An algorithm for appropriate flap choice. Medicine (Baltimore) 98 (2019): e18021. 31725677 10.1097/MD.0000000000018021 PMC6867784

[74] Chen J, Costello CM, Mead-Harvey C, Full-thickness skin grafts in nasal reconstruction: A retrospective study. JAAD Int 13 (2023): 91–94. 37752938 10.1016/j.jdin.2023.08.004 PMC10518335

[75] Kasem AT, Sakrana AA, Ellayeh M, Evaluation of zirconia and zirconia-reinforced glass ceramic systems fabricated for minimal invasive preparations using a novel standardization method. J Esthet Restor Dent 32 (2020): 560–568. 32011094 10.1111/jerd.12570

[76] Dumont S, Msangi S, Ponthus S, Health-Related Quality of Life and Social Reintegration Indicators Following Reconstructive Surgery: A Prospective Observational Study. World J Surg 49 (2025): 2794–2800. 40635168 10.1002/wjs.12696 PMC12515029

[77] Lee J, Lee J, Hong YS, Impact of tumor size by clinical N subclassification and histology in trimodality-treated N2 non-small cell lung cancer. Sci Rep 15 (2025): 17195. 40382370 10.1038/s41598-024-82946-y PMC12085672

[78] Grigoriadis A, Gazinska P, Pai T, Histological scoring of immune and stromal features in breast and axillary lymph nodes is prognostic for distant metastasis in lymph node-positive breast cancers. J Pathol Clin Res 4 (2018): 39–54. 29416876 10.1002/cjp2.87 PMC5783956

[79] Patkar S, Chen A, Basnet A, Predicting the tumor microenvironment composition and immunotherapy response in non-small cell lung cancer from digital histopathology images. NPJ Precis Oncol 8 (2024): 280. 39702609 10.1038/s41698-024-00765-w PMC11659524

[80] Erdag G, Schaefer JT, Smolkin ME, Immunotype and immunohistologic characteristics of tumor-infiltrating immune cells are associated with clinical outcome in metastatic melanoma. Cancer Res 72 (2012): 1070–1080. 22266112 10.1158/0008-5472.CAN-11-3218 PMC3306813

[81] S Chou J W, Willingham MC, Ruiz J, Interactions between immunity, proliferation and molecular subtype in breast cancer prognosis. Genome Biol 14 (2013): R34. 23618380 10.1186/gb-2013-14-4-r34 PMC3798758

[82] Shui Y, Li M, Su J, Prognostic and clinicopathological significance of systemic immune-inflammation index in pancreatic cancer: a meta-analysis of 2,365 patients. Aging (Albany NY) 13 (2021): 20585–20597. 34435973 10.18632/aging.203449 PMC8436945

[83] Williams GJ, Quinn T, Lo S, Mohs micrographic surgery for the treatment of invasive melanoma: A systematic review with meta-analyses. J Eur Acad Dermatol Venereol 39 (2025): 416–425. 38842170 10.1111/jdv.20138 PMC11760679

[84] Chen M, Yunqiong Wang Y. Survival Outcomes of Mohs Surgery versus Wide Local Excision for Less Common Nonmelanoma Skin Cancers: A Stabilized Inverse Probability of Treatment Weighting Analysis. Dermatology 239 (2023): 877–888. 37699383 10.1159/000533350

[85] Cheraghlou S, Christensen SR, Agogo GO, Comparison of Survival After Mohs Micrographic Surgery vs Wide Margin Excision for Early-Stage Invasive Melanoma. JAMA Dermatol 155 (2019): 1252–1259. 31553403 10.1001/jamadermatol.2019.2890 PMC6764120

[86] Shah P, Trepanowski N, Grant-Kels JM, Mohs micrographic surgery in the surgical treatment paradigm of melanoma in situ and invasive melanoma: A clinical review of treatment efficacy and ongoing controversies. J Am Acad Dermatol 91 (2024): 499–507. 38768857 10.1016/j.jaad.2024.05.024

[87] Bosdriesz JR, Stel VS, van Diepen M, Evidence-based medicine-When observational studies are better than randomized controlled trials. Nephrology (Carlton) 25 (2020): 737–743. 32542836 10.1111/nep.13742 PMC7540602

[88] Gale RP, Zhang MJ, Lazarus H M The role of randomized controlled trials, registries, observational databases in evaluating new interventions. Best Pract Res Clin Haematol 36 (2023): 101523. 38092482 10.1016/j.beha.2023.101523

[89] Jones CW, Keil LG, Holland WC, Comparison of registered and published outcomes in randomized controlled trials: a systematic review. BMC Med, 13 (2015): 282. 26581191 10.1186/s12916-015-0520-3 PMC4650202

[90] Kubesch N, Gaitonde S, Petriti U, Use cases of registry-based randomized controlled trials-A review of the registries’ contributions and constraints. Clin Transl Sci 17 (2024): e70072. 39558508 10.1111/cts.70072 PMC11573736

[91] Petrisor B, Bhandari M. The hierarchy of evidence: Levels and grades of recommendation. Indian J Orthop 41 (2007): 11–15. 21124676 10.4103/0019-5413.30519 PMC2981887

[92] Speich B, Gloy VL, Klatte K, Reliability of Trial Information Across Registries for Trials With Multiple Registrations: A Systematic Review. JAMA Netw Open 4 (2021): e2128898. 34724557 10.1001/jamanetworkopen.2021.28898 PMC8561329

[93] Walker KF, Stevenson G, Thornton JG. Discrepancies between registration and publication of randomised controlled trials: an observational study. JRSM Open 5 (2014): 2042533313517688. 25057391 10.1177/2042533313517688 PMC4012655

[94] Wandalkar P, Gandhe P, Pai A, A study comparing trial registry entries of randomized controlled trials with publications of their results in a high impact factor journal: The Journal of the American Medical Association. Perspect Clin Res 8 (2017): 167–171. 29109933 10.4103/2229-3485.215978 PMC5654215

[95] Cheraghlou S, Doudican NA, Criscito MC, Overall Survival After Mohs Surgery for Early-Stage Merkel Cell Carcinoma. JAMA Dermatol 159 (2023): 1068–1075. 37610773 10.1001/jamadermatol.2023.2822 PMC10448369

[96] Murray C, Sivajohanathan D, Hanna TP, Patient indications for Mohs micrographic surgery: a clinical practice guideline. Curr Oncol 26 (2019): e94–e99. 30853814 10.3747/co.26.4439 PMC6380643

[97] Orr LD, Vanderpoel J, Vadagam P, Patient, care partner, and provider voice in treatment decision-making for non-small cell lung cancer. Patient Educ Couns 136 (2025): 108776. 40215576 10.1016/j.pec.2025.108776

[98] Thompson KG, Manoharan D, Tripathi R, Predictors of patient satisfaction with Mohs micrographic surgery at time of surgery and 3 months postsurgery: A prospective cohort study. J Am Acad Dermatol 89 (2023): 992–1000. 37422015 10.1016/j.jaad.2023.06.046

[99] Catalan-Griffiths A, Pasquali P, Arias-Santiago S, Shared decision-making quality and decisional regret in patients with low-risk superficial basal cell carcinoma: A prospective, multicenter cohort study. JAAD Int, 13 (2023): 159–163. 37823045 10.1016/j.jdin.2023.05.015 PMC10562145

[100] Vaidya TS, Bander TS, Musthaq S, Validation of a patient decision aid for the treatment of lentigo maligna. J Am Acad Dermatol 84 (2021): 1751–1753. 33470203 10.1016/j.jaad.2020.10.043 PMC8406223

[101] Maciejewska M, Betkowska A, Czuwara J, Mohs Micrographic Surgery: A Narrative Review of Current Practices, Emerging Trends, and Case-Based Insights. Adv Ther 42 (2025): 5397–5426. 40965782 10.1007/s12325-025-03354-w PMC12579696

[102] Levy JJ, Davis MJ, Chacko RS, Intraoperative margin assessment for basal cell carcinoma with deep learning and histologic tumor mapping to surgical site. NPJ Precis Oncol 8 (2024): 2. 38172524 10.1038/s41698-023-00477-7 PMC10764333

[103] Schneider SL, Kohli I, Hamzavi IH, Emerging imaging technologies in dermatology: Part II: Applications and limitations. J Am Acad Dermatol 80 (2019): 1121–1131. 30528310 10.1016/j.jaad.2018.11.043 PMC7469877

[104] Burke O, Hartoyo M, Lin R, The financial impact and utilization of inpatient dermatology services: historical insights and future implications. Arch Dermatol Res 317 (2025): 374. 39921720 10.1007/s00403-025-03867-y PMC11807067

[105] Jain NP, Gronbeck C, Beltrami E, Mohs Surgery Price Transparency and Variability at Academic Hospitals After the Implementation of the Federal Price Transparency Final Rule. JMIR Dermatol 6 (2023): e50381. 37966874 10.2196/50381 PMC10687679

[106] Sampath AJ, Paci K, Carrasquillo OY, Retrospective analysis shows the cost of Mohs surgery decreases when adjusted for medical inflation. J Am Acad Dermatol 89 (2023): 1001–1006. 37422019 10.1016/j.jaad.2023.06.041

[107] Rogers HW, Coldiron B M A relative value unit-based cost comparison of treatment modalities for nonmelanoma skin cancer: effect of the loss of the Mohs multiple surgery reduction exemption. J Am Acad Dermatol 61 (2009): 96–103. 19539843 10.1016/j.jaad.2008.07.047

